# Road-traffic pollution and asthma – using modelled exposure assessment for routine public health surveillance

**DOI:** 10.1186/1476-072X-3-24

**Published:** 2004-10-14

**Authors:** Elspeth C Ferguson, Ravi Maheswaran, Mark Daly

**Affiliations:** 1 Public Health GIS Unit, School of Health and Related Research, The University of Sheffield, Regent Court, 30 Regent Street, Sheffield S1 4DA, United Kingdom; 2 Environmental Protection Service, Sheffield City Council, Environment and Regulatory Services, 2-10 Carbrook Hall Road, Sheffield S9 2DB, United Kingdom

## Abstract

Asthma is a common disease and appears to be increasing in prevalence. There is evidence linking air pollution, including that from road-traffic, with asthma. Road traffic is also on the increase. Routine surveillance of the impact of road-traffic pollution on asthma, and other diseases, would be useful in informing local and national government policy in terms of managing the environmental health risk.

Several methods for exposure assessment have been used in studies examining the association between asthma and road traffic pollution. These include comparing asthma prevalence in areas designated as high and low pollution areas, using distance from main roads as a proxy for exposure to road traffic pollution, using traffic counts to estimate exposure, using vehicular miles travelled and using modelling techniques. Although there are limitations to all these methods, the modelling approach has the advantage of incorporating several variables and may be used for prospective health impact assessment.

The modelling approach is already in routine use in the United Kingdom in support of the government's strategy for air quality management. Combining information from such models with routinely collected health data would form the basis of a routine public health surveillance system. Such a system would facilitate prospective health impact assessment, enabling policy decisions concerned with road-traffic to be made with knowledge of the potential implications. It would also allow systematic monitoring of the health impacts when the policy decisions and plans have been implemented.

## Introduction

The prevalence of asthma is increasing and there is concern that the increase may in part be attributable to increasing road traffic related pollution. The concerns relate especially to childhood asthma. In this article, we set out the arguments for using modelled exposure assessment to create a surveillance system that will facilitate routine public health work, such as monitoring and health impact assessment. We first discuss the increasing prevalence of asthma and the effects of air pollution on asthma. We then set out the benefits of monitoring the link between outdoor air pollution related to road traffic and asthma and discuss methods of exposure assessment in some detail. We describe modelling air quality in the UK as an example and then address the implementation of a surveillance system.

### Increasing prevalence of asthma

Asthma is a common disease. Figures from the International Study of Asthma and Allergies in Childhood suggest up to 25.9% of children in Oceania have ever had asthma [1]. Although less evidence is available with regard to the prevalence amongst adults, the European Community Respiratory Health Study which studied asthma prevalence throughout Europe, Australasia and the United States suggested asthma affects up to 11.9% of adults in Australia and 8.4% of adults in the UK [2]. Over recent years the prevalence of asthma appears to have been steadily increasing [3]. It has also been suggested by some that the severity of asthma is on the increase [4], although other studies do not confirm this suggestion [5]. Initially thought to be a disease of the western world, in recent years the incidence of asthma has also been shown to be increasing dramatically in less developed countries [6]. Much research has taken place to find the cause of such an increase but the reasons are not fully understood. Socio-economic status [7], ethnicity [8], allergen exposure [9], smoking [10], nutrition [11] and infection exposure [12] have all been considered as possible factors. Links have also been made to living in an urban as opposed to a rural area [13], raising speculation as to a possible effect of air pollution on the prevalence of the disease.

### Effects of air pollution on asthma

Air pollution has been linked to morbidity and mortality of several diseases, including diverse conditions such as coronary heart disease [14] and Hodgkin's disease [15]. In terms of the effects on respiratory disease, exposure to air pollution has been linked to the aggravation of chronic respiratory symptoms and increased mortality from chronic obstructive pulmonary disease [16]. Over the 20^th ^century, air pollution increased greatly alongside the noted increase in the prevalence of asthma. Yet the last decades of the century have seen a considerable reduction in pollutants such as sulphur dioxide (SO_2_) following a cut back on industrial emissions [17].

Levels of pollutants such as nitrogen dioxide (NO_2_) however, still remain problematic due to the increasing number of vehicles on our roads. In fact almost 50% of NO_2 _is thought to be produced by vehicles and much particulate matter is produced by diesel exhaust fumes [17]. Experimental studies have shown NO_2 _exposure increases cell membrane permeability, decreases ciliary beat frequency [18] and increases the response of asthmatics to inhaled allergens [19], whilst exposure to diesel exhaust particles in mice has been shown to alter IgE antibody production [20]. Epidemiological investigations into the effects of these pollutants have suggested an association between pollutant levels and the exacerbation of asthma. Results from a study in Paris showed an increase of 100 μg/m^3 ^of NO_2 _to be associated with a relative risk of 1.175 for asthma admission [21]. Emissions of nitrogen oxides (NO_x_) have also been shown to influence emergency room visits in Israel [22]. Both particulate matter less than 10 μm in diameter (PM_10_) and NO_2 _appeared to consistently increase attendances at accident and emergency units with asthma in London [23]. With traffic emissions accounting for such a high proportion of these pollutants and with the volume of traffic on the increase, such a link between traffic-related pollution and asthma would be of importance.

It has been suggested that pollutant exposure may induce asthma, aggravate asthma or increase the permeability of the airways to other allergens to which asthmatics are susceptible [17]. Any of these effects could potentially cause a significant increase in asthma morbidity if the level of traffic continues to rise. A number of studies have considered the association between asthma and road-traffic pollution specifically. Ciccone et al. [24] showed the odds ratios for asthma and a number of asthmatic symptoms to be increased in those exposed to heavy lorry traffic. Heavy traffic flow has also been shown to increase childhood asthma admissions [25]. Studies carried out within the UK and the United States have suggested those living within close proximity to a road are also at an increased risk of hospitalisation with asthma [25,26].

### Monitoring the link between air pollution and asthma

The evidence suggests that there is a link between air pollution and asthma but it is not conclusive. The increasing prevalence of asthma and the continuing increase in road traffic are both of concern. Monitoring the association between asthma and road traffic pollution would be useful for public health purposes, both in terms of surveillance and in terms of influencing policy. Policy implications might include routing of traffic, construction of bypasses, congestion reduction schemes, utilisation of non-fossil fuel cars and possibly even the location of schools. A monitoring system would use estimates of air pollution from road traffic which would be linked to data on asthma obtained from routine systems, such as hospital admissions, attendance at accident and emergency departments or primary care consultations or to data from periodic surveys on health, including asthma prevalence.

### Methods of exposure assessment

A number of methods for exposure assessment have been used in studies examining the association between asthma and road traffic pollution and these are described below. These include comparing asthma prevalence in areas designated as high and low pollution areas, using distance from main roads as a proxy for exposure to road traffic pollution, using traffic counts to estimate exposure, using vehicular miles travelled and using modelling approaches which can take into account a number of variables.

#### (i) High vs low pollution areas

One method frequently used is estimating exposure levels of individuals based on assessing whether a residence is in a high or low pollution area. Many have estimated exposure status of individuals on the basis of whether or not they live on a street with heavy traffic, for example in the study conducted by Jedrychowski and Flak [27]. Another approach was used by Nicolai and v. Mutius [28] in their study of former East and West Germany. In this study, West Germany was classified as an area with high traffic-related NO_2 _emissions and low SO_2 _emissions from industry, whilst the opposite was said to be true of East Germany. The problem, particularly with this method, is that although the countrywide generalisation may hold true, it may not be true for each individual. Even when the measure is made for each individual, it should be remembered it may be subject to bias. It has been postulated that asthmatic individuals and their families may be more aware of the speculation over such a link between road-traffic and asthma and therefore may be more likely to report or consider heavy traffic to be associated with their symptoms [24].

#### (ii) Distance to roads

Distance from roads has commonly been used as a proxy for road traffic exposure in a number of studies. Postcodes are georeferenced and may be used in a geographical information system (GIS) to calculate the distance from an individual's residence to a road, most often a main road, carrying over a certain volume of vehicles. In certain cases the distance from a child's school to a main road has been used instead. Examples of the use of this method are studies by Livingstone et al. [29] and Wilkinson et al. [30]. A number of authors have only considered individuals living within 1000 m of a main road as they felt traffic would be unlikely to influence pollution levels beyond this distance [31]. Indeed, with analyses using this method, effects of pollutants have often only shown an effect within a short distance from main roads. By using this method an assumption is made that all individuals living within a certain distance of a road are subjected to the same level of exposure, yet this is unlikely to be the case. Traffic on different roads varies, both in volume and in type, and meteorological conditions can alter dispersion of pollutants. It is well known that cold weather conditions trap air close to the ground, prolonging the duration of the time pollutants remain close to where they were produced [17].

#### (iii) Traffic counts

Another popular method is considering traffic flow along the street of residence or one in close proximity, as used by English et al. [32]. In a similar way Wjst et al. [33] have investigated traffic flow around a child's school. The traffic count method has the advantage of being likely to be a more valid measure than distance to roads. It is worth however considering the daily movements of an individual. Throughout the day an individual travels between home and work or school experiencing a number of different exposure levels on the way. Recreational activities may also subject a person to different levels of exposure. Indeed even within the residential area, exposure may vary dependent on the time one spends indoors or outdoors. Indoor exposure to NO_2 _may be high, with levels possibly higher than outdoors if a gas stove is used in the home [34]. Another point to consider is the type of traffic exposure. Emissions vary greatly between cars and trucks. Some have approached this by analysing data from different vehicles separately, suggesting truck pollution to be more detrimental to health than that from cars [31]. It could be suggested that as car and truck pollution varies, for example, trucks produce a lot more particulate matter consisting of diesel particles than cars, that perhaps one should consider the effects of particulates separately from those of NO_2_. This however, is not without difficulties, as if one is exposed to traffic there will be a combined effect from a cocktail of pollutants produced by both trucks and cars.

#### (iv) Vehicle miles travelled

Some authors (e.g. Lin et al. [26]) have attempted to combine both length of road and traffic counts as a measure referred to as vehicle miles travelled. It involves multiplying the length of a road in a specified area around the home by the traffic volume travelling along that section of road. Authors have varied in the selection of roads used in such analyses. This method may have an advantage over measuring traffic flow alone, as exposures may be more accurate. There is still however, no account taken of individuals moving between areas through the day or different topographical conditions. Some feel that buildings in the vicinity of one's home should be considered when looking at exposure to traffic pollution [35], due to their influence on pollution dispersion, as well as the presence of bus stops and distance to street crossings which may influence exposure [27].

#### (v) Modelling approach

A number of studies have used modelling to estimate pollution exposure [35,36]. A model is capable of taking into account a whole range of factors that may affect exposure. As illustrated by Pershagen et al. [36] exposures both at home and at day-care centres or for others at school or work can be considered, with these being adjusted for the time spent in each location. Factors considered in the models used in the studies above have included vehicle type and density, presence and type of buildings on a street, meteorological conditions, street width and distance from house to middle of the street amongst other factors.

Even within a model however, accounting for personal day-to-day exposures is still problematic. In order to take previous exposures into account a cohort study would be necessary [37]. Certainly if one is trying to account for the prevalence of a disease like asthma, knowing previous exposure levels prior to the onset of the disease is important. To do this one would need to look at the previous residences and day-to-day exposures of that person throughout their life. An alternative would be to use a personal monitoring system. Both these methods of assessing long-term exposure, however, would be very expensive. One could consider the use of monitoring stations already in place throughout cities. The problem with using such stations is that they are generally widely dispersed while pollution levels may vary substantially within short distances, e.g. exponential decline in the concentration of certain pollutants with increasing distance from busy roads [38]. Installing sufficient monitoring stations to adequately capture spatial variation in levels of pollution encountered over short distances, would be both impractical and expensive.

Despite limitations, a model would appear to be the most practical way of assessing traffic-related exposure where routine surveillance is concerned. Information such as vehicle density, type of vehicle, risk of traffic congestion, presence of bus stops and street crossings, distance of residences to roads, street width, type of street, building presence and type and meteorological conditions (e.g. wind speed and direction, absolute temperature and temperature differences, global and gamma radiation) could be collected routinely for use in a variety of models for predicting exposure to NO_2 _and PM_10_. The model could be used to estimate exposures on all the streets within a certain radius of the home or place of work as dispersion of pollutants from these streets may also be affecting the individual. In a sophisticated model it may be possible to make adjustment for the height of an individual's residency or place of work in high rise buildings to account for the vertical dispersion of pollutants. Such a system could also be used to estimate exposures at previous residences, work places or schools of an individual so that an assessment of lifelong exposure could be made as accurately and practically as possible. However, the latter might be too complicated for a routine monitoring system.

### Modelling air quality in the UK

The UK Government's current policy on air quality within the UK is set out in the Air Quality Strategy for England, Scotland, Wales and Northern Ireland published in January 2000 pursuant to the requirements of Part IV of the Environment Act 1995. The Strategy sets out a framework for improving air quality and for ensuring that international commitments are met. It is designed to be an evolving process that is monitored and regularly reviewed. The Strategy sets standards and objectives for ten pollutants that have an adverse effect on human health, vegetation or ecosystems and target dates for achieving them. The standards generally set concentration limits above which sensitive members of the public (e.g. children, older people, people who are unwell) might experience adverse health effects. In early 2003 an Addendum to the Strategy was published introducing standards and objectives for a new pollutant and revising those for three others.

The pollutants currently specified in the Strategy now include benzene, 1,3 butadiene, carbon monoxide, lead, NO_2_, PM_10_, SO_2_, ozone (O_3_), NO_x _and polycyclic aromatic hydrocarbons. The predominant source of most of these pollutants is road traffic, but industrial and domestic sources are also contributors.

The air quality standards or guideline limits are long-term benchmarks for ambient pollutant concentrations which represent negligible or zero risk to human health, based on medical and scientific evidence reviewed by the Expert Panel on Air Quality Standards (EPAQS) and the World Health Organization (WHO). For some pollutants, (e.g. NO_2_), there is both an annual mean guideline limit and a short-term mean guideline limit. These reflect the varying impacts on human health of exposure to some pollutants over differing time periods, (e.g. temporary exposure on the pavement adjacent to a busy road compared with the exposure of residential properties adjacent to a road).

The air quality objectives are medium-term policy-based targets set by the Government which take into account economic efficiency, practicability, technical feasibility and timescale. Some objectives are equal to the EPAQS or WHO recommended air quality standards and guideline limits, whereas others involve a margin of tolerance, i.e. a limited number of permitted exceedances of the standard over a given period.

The Government has issued guidance to local authorities on how to conduct Reviews and Assessments required under the system of Local Air Quality Management (LAQM). The latest available guidance is Policy Guidance LAQM.PG(03) and Technical Guidance LAQM.TG(03). Air quality modelling is key to assessing the future potential for attainment, or not, of the objectives. Part IV of the Environment Act 1995 requires a local authority to designate an Air Quality Management Area (AQMA) covering any part of its administrative area where air quality objectives are not likely to be achieved by, or at any point beyond, the relevant objective's target date at locations where the general public might reasonably be exposed. These AQMAs have been determined by modelling future scenarios. For each AQMA the local authority has a duty to draw up an Air Quality Action Plan (AQAP) setting out the measures the authority intends to introduce to deliver improvements in local air quality in pursuit of the air quality objectives. Local authorities are not statutorily obliged to meet the objectives, but they must show that they are working towards them. As of June 2004, there were 120 designated AQMAs in the UK, with 80 AQAPs produced outlining how air quality would be tackled in these areas.

### Implementation of a surveillance system

A routine surveillance system would be one which links modelled air quality data, such as those derived in the UK described above, with routinely collected health data. There are a number of issues regarding the technical aspects of linking spatial information on air quality with health information, typically carried out using GIS. These are discussed in detail elsewhere [39]. The information on air quality could be used in a statistical model and analysed alongside hospital admission, accident and emergency attendance and prevalence data for asthma, or any other conditions to which a link with air pollution has either been made or considered.

The majority of studies examining the link between asthma and road-traffic pollution have concentrated on the prevalence of asthma, for example studies by Ciccone et al. [24], Jedrychowski and Flak [27], Livingstone et al. [29] and Nicolai and v. Mutius [28]. Prevalence data is typically gathered from health surveys, as information regarding disease prevalence is not generally available through routine recording. It would however be possible through the use of periodic health surveys to collect the relevant information required to analyse disease prevalence along with modelled pollution exposure data. The ISAAC investigators for example have designed a standardised questionnaire now being used throughout the world which is concerned with the prevalence of asthma amongst children. It considers those children suffering from asthma to be those who answer, or whose parents answer, that they have suffered from wheezing or whistling from the chest in the last twelve months [40]. Using a standardised questionnaire such as the ISSAC questionnaire for children would allow comparable information to be collected and compared both within and between countries.

A few of the studies examining the link between asthma and road-traffic pollution have used information that is readily available. For example studies by English et al. [32] and Lin et al. [26] have looked at children being hospitalised with exacerbations of asthma. Such information is routinely recorded in hospitals, but tends to reflect disease severity. Relating such information to levels of air pollution is still important in determining the effects of pollution on asthma.

A routine surveillance system recording spatial variation in pollutant levels would allow improved understanding of the link between road-traffic pollution and asthma, or indeed other diseases and could be used to help predict future health impact, particularly in cities and towns. The results of such assessment would allow local policy decisions concerning the routing of traffic around residential areas or schools and plans to reduce congestion to be made with knowledge of the implications of the decision on the health of the local population. It would also allow systematic monitoring of the health impacts when the policy decisions and plans have been implemented.

We should point out here that to examine acute effects (i.e. on events such as admissions, general practice consultations, emergency room attendances) using daily time series analyses, monitoring data would be required as modelling would probably be too insensitive to detect daily variation in outdoor air pollution levels. However, time series analyses are complicated research methods that are not within the realm of routine public health practice. What we have argued for here is a surveillance system that looks at spatial variation in pollution and asthma which would highlight problem areas and could monitor the effects of interventions to reduce pollution in these areas.

## Conclusions

We believe that a routine surveillance system which links modelled outdoor air pollution data to health data would provide a useful tool for facilitating routine environmental public health work. Such a system would be especially useful for monitoring the health effects of traffic related pollution and for aiding health impact assessment. Implementation of the system will require close collaboration between public health and environmental health departments, protocols for sharing data and investment in training to develop the necessary technical expertise to set up and maintain the surveillance system. Of particular importance will be the ability of high level management to interpret surveillance information within a wider policy context.

## Authors' contributions

RM proposed the idea for the article. ECF wrote the first draft, supervised by RM. MD contributed the section on modelling air quality in the UK. RM edited subsequent drafts.
